# A New and Reliable Guide for Studies of Neuronal Loss Based on Focal Lesions and Combinations of *In Vivo* and *In Vitro* Approaches

**DOI:** 10.1371/journal.pone.0060486

**Published:** 2013-04-09

**Authors:** Vera Paschon, Guilherme Shigueto Vilar Higa, Lais Takata Walter, Érica de Sousa, Fausto Colla Cortesão Zuzarte, Vivian Roca Schwendler Weber, Rodrigo Ribeiro Resende, Alexandre Hiroaki Kihara

**Affiliations:** 1 Núcleo de Cognição e Sistemas Complexos, Centro de Matemática, Computação e Cognição, Universidade Federal do ABC, Santo André, São Paulo, Brasil; 2 Departamento de Fisiologia e Biofísica, Instituto de Ciências Biomédicas, Universidade de São Paulo, São Paulo, São Paulo, Brasil; 3 Departamento de Bioquímica e Imunologia, Instituto de Ciências Biológicas, Universidade Federal de Minas Gerais, Belo Horizonte, Minas Gerais, Brasil; Nathan Kline Institute and New York University School of Medicine, United States of America

## Abstract

In this study, we describe a simple and reliable method to study neuroprotective effects in living and organized neural tissue. This method, which was based on retinal explants for *in vivo* focal lesions, was conceived as a collection of modular procedures, which can be customized for particular demands. With this model, it is possible to combine immunohistochemistry with image data analysis to track the two- or three-dimensional redistribution of proteins as a time/space function of primary cell loss. At the same time, it is possible to finely control the exposure of the tissue to specific drugs and molecules. In order to illustrate the use of the proposed method, we tested the effects of two different nanotube compounds on retinal explant viability. Transcriptome analyses can be separately performed in the lesion focus and *penumbra* with laser capture microdissection followed by polymerase chain reaction analyses. In addition, other common experimental drawbacks, such as high individual variance, are eliminated. With intraocular injections, treatments can be verified *in vivo*, with one eye serving as the experimental tissue and the other serving as the control tissue. In summary, we describe a flexible and easy method, which can be useful in combination with a broad variety of recently developed neuroprotective strategies, to study neurodegeneration.

## Introduction

Molecular and cellular therapies that aim to minimize apoptotic effects reveal potential new scenarios for the treatment of neurodegenerative diseases. From nucleic acid aptamers and microRNA (miRNA) antagomirs to the specific delivery controls that are provided by carbon and peptide nanotubes, investigations that use specific concentrations and combinations of molecules and are performed *in vivo* are often comprehensive, particularly considering individual variability. However, neuronal and glial cell cultures offer the convenience of *in vitro* models, but the disruption of the original synaptic networks and the lack of an extracellular matrix environment prevent conclusions about the data from a physiological perspective [1].

When a neurodegenerative process is triggered, several mechanisms result in secondary cell death, including changes in the concentration of extracellular ions, the release of free oxygen radicals, energy depletion, high levels of the excitatory neurotransmitter glutamate, altered intracellular calcium homeostasis, and the regulation of gene expression [2], [3], [4]. Different methods have been proposed to inhibit apoptosis spread, and these could provide efficient strategies for the treatment of stroke, Alzheimer’s disease, Parkinson’s disease, and other neuronal diseases [5]. Monoclonal antibodies and oligonucleotide therapeutics, such as antisense and small interfering RNA, represent excellent tools for validating targets by functional inactivation of specific protein activity or by knocking out gene expression [6]. New approaches that aim to control unbalanced transcriptomes and/or proteomics, such as nucleic acid aptamers and miRNA antagomirs, have been developed [7], [8]. In addition, the efficacy of drug delivery and its combination with nanotechnology, such as carbon and peptide nanotubes, have been extensively studied [9], [10].

The retina is a highly organized and easily accessible part of the central nervous system. It has a clear laminar structure and a considerable variety of cell types. Therefore, it is considered a natural brain slice, and an attractive model to study the central nervous system [11]. Moreover, the vitreous chamber acts as a capsule for drug delivery to the retina, permitting experimental manipulations through *in vivo* intraocular injections [12]. However, *in vitro* studies have many advantages, including the ability to highly control conditions, which allow for measurements on a cell-by-cell basis, isolation from confounding systemic effects, time course flexibility, and a reduction in the number of animals required for the research. However, possible limitations include the selective loss of specific cell phenotypes/functions, changes in tissue architecture, and the questionable relevance of *in vitro* findings. The maintenance of preserved tissue and its original architecture and extracellular matrix provides a more realistic physiological interpretation. In this regard, the use of retinal organotypic cell cultures, which are also known as retinal explants, could be a great option because they retain many histological and biochemical features and can be maintained *in vitro* for several days or even weeks [1].

**Figure 1:**
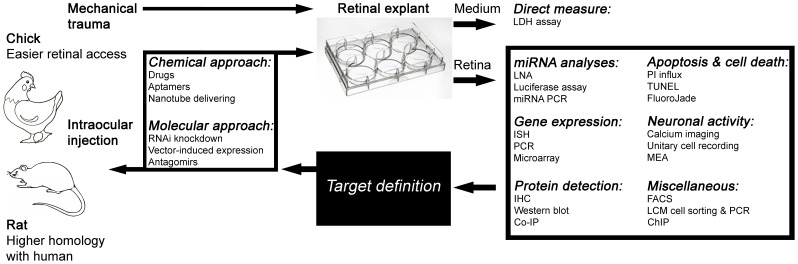
Summary of the proposed guide for neurodegeneration studies with a retinal trauma model. Retinal lesions can be produced with a thin needle in different species, including chicks and rats. Chick retina offers easy access because of its size, even in embryonic ages, whereas experiments that are conducted in rats may be of clinical relevance due to their higher homology with humans. After different post-lesion time points, the animals were euthanized, and the retinas were utilized in different methods. Retinal explants can be treated with different classes of molecules, including aptamers, antagomirs, or carbon nanotubes. The lactate dehydrogenase (LDH) concentration in the culture medium can be used as a reliable probe of cell viability. Afterwards, the retinas can be utilized in a wide range of experimental analyses, from epigenetics, such as microRNA (miRNA) activity to chromatin alterations, and live cell activity recording, which employs calcium imaging or multielectrode arrays. Once the role of a particular target molecule or gene is determined during apoptosis progression, *in vivo* validation can be performed by comparing control *vs.* experimental conditions in the same animal. The use of both eyes from the same animal allows for simple statistical analyses such as paired *t*-tests.

The retina offers an exceptional model to study trauma-induced cell death because of its easy accessibility and structural uniformity, which allows for site-restricted injuries and the reliable quantification of cellular damage [13]. As shown herein, we describe an easy and simple method that is based on mechanical trauma caused by a thin needle, resulting in precise definition of the lesion site, without global traumatization observed in ischemia models [14], [15]. Moreover, whole retinas may be dissected after different time points, enabling reliable quantitative comparisons of miRNA, gene expression, and protein concentrations. When combined with other techniques, such as lactate dehydrogenase (LDH) assays [16], laser capture microdissection (LCM) [17], [18], or terminal deoxynucleotidyl transferase (TdT)-mediated 2-deoxy-uridine-5-triphosphate (dUTP) nick-end labeling (TUNEL) [19], this method greatly reduces the individual variance and can be customized for particular demands. In addition to analyses of the explant medium, several methodologies can be applied to the retinal explant itself, including histological examinations after specific treatments. In Fig. 1, we present an overall view of the possibilities that can be performed using the described method.

Although some of the procedures presented herein have been previously described, the combination of these techniques with mechanical lesions of the retina is described for the first time in this study as a general guide to evaluate neuronal loss.

## Methods

### Ethics Statement

The experiments were conducted in accordance with guidelines of the National Institutes of Health and the Brazilian Scientific Society for Laboratory Animals. The experimental protocol was approved by the Ethics Committee in Animal Experimentation of the Institute of Biomedical Sciences/University of São Paulo (ICB/USP).

### Animal Procedures

In this study, we utilized male chicks (*Gallus gallus*) that were 7–15 days of age and rats (*Rattus novergicus*) that were 50–60 days of age. All animals were bred and housed in a vivarium with free access to food and water and were kept on a 12∶12-h light/dark cycle with lights on at 06∶00 a.m. Chick colonies were maintained at 32–35°C in the first week and 29–32°C and 26–29°C in the second and third weeks, respectively. However, rats were maintained constantly at 22–24°C. Chicks were fed CN Frango Inicial (20% protein, CN Rações, Bom Jardim, RJ, Brazil), and rats were fed Nuvilab CR-1 (23% protein, Quimtia Brasil, Colombo, PR, Brazil) Animals were not handled before the lesion procedure, except for cage cleaning.

Animals were anesthetized with ketamine (10 mg/100 g of body weight, i.p., Parke-Davis, Ann Arbor, MI, USA) and xylazine (1 mg/100 g of body weight, i.p., Miles Inc., West Haven, CT, USA). In order to confirm that the animals were anesthetized, a paw was pressed to test the pain withdrawal reflex. Thereafter, animals were subjected to 6 local mechanical lesions in the retina. The lesions were made with a thin needle (28-gauge, 12.7 mm, BD Medical–Diabetes Care, Franklin Lakes, NJ, USA), which crossed the cornea, lens, vitreous, and retina. The lesions were performed without completely removing the needle from the eye in order to cause minimal damage to the cornea. After different periods, animals were euthanized with an overdose of ketamine (30 mg/100 g, i.p.) and xylazine (2 mg/100 g, i.p.). The eyecups were removed, and the retinas were gently dissected for different methodologies.

### Immunohistochemistry

Seven-day-lesioned chick eyes were fixed for 30 min in 4% paraformaldehyde (PFA, Sigma-Aldrich Co. LLC, St. Louis, MO, USA) diluted at <60°C in phosphate-buffered saline (PBS; 0.1 M, pH 7.3) and cryoprotected in 30% sucrose solution for 24 h at 4°C. After embedding the eyes in Optimum Cutting Temperature compound (OCT, Sakura Finetek USA, Inc., Torrance, CA, USA), they were cut transversally (12 µm) on a cryostat, and 1 in 5 sections were sampled from the nasal to the temporal direction. For the analysis, we examined 3–5 sections in each animal (n = 3). Retinal sections were blocked for 30 min in a solution containing 10% normal goat serum, 1% bovine serum albumin, and 0.3% Triton-X 100 in PBS.

To characterize the reactive gliosis in the retinal lesion, a mouse monoclonal antibody that was raised against glial fibrillary acidic protein (GFAP) was employed (#G3893, 1∶4,000, Sigma-Aldrich Co. LLC) in order to identify Müller glial cells, as described in other studies [20]. Controls for the experiments consisted of the omission of primary antibodies; no staining was observed in these cases. After several washes, retinal sections were incubated with goat antiserum against mouse IgG that was tagged to Alexa TM 488 (Life Technologies Corporation, Grand Island, NY, USA 1∶250–1∶500) and diluted in PBS containing 0.3% Triton X-100 for 2 h at room temperature. Retinas were incubated with propidium iodide (1∶4,000) for 10 min and washed thrice in PBS for 5 min. Finally, sections were coverslipped with Vectashield Mounting Media (Vector Laboratories, Inc., Burlingame, CA, USA).

### Image Analysis

Slides were analyzed with a Nikon TS100F inverted microscope, and photomicrographs were acquired with NIS-elements AR 3.2 64-bit software (Nikon Instruments Inc., Melville, NY, USA). Image analysis was performed with Image-Pro Plus software (Media Cybernetics, Inc., Bethesda, MD, USA), as previously described [21]. In summary, after channel separation (RGB) of the color images, we performed two different analyses: (i) counting cells/nuclei: after proper setting of the size and brightness, the software identified discrete elements and automatically counted labeling that was displayed in the image, and thus, artifacts and background labeling could be identified and discarded and (ii) bitmap analysis: *x–y* axis analyses generated numerical-appended data files corresponding to the pixel values. The bitmap analysis was used to view the pixel values of the active window (or area of interest) in a numerical format, in which the values corresponded to the brightness of the pixels. This matrix was exported to Excel (Microsoft, Redmond, WA, USA) for appropriate mathematical computations. The numerical data generated a histogram, which essentially averaged the labeling intensities at different retinal locations. Photomicrographs and charts were prepared with Adobe Photoshop CS2 (Adobe Systems Inc., San Jose, CA, USA).

### Carbon Nanotube Synthesis

Single-wall carbon nanotubes (SWCNTs) were prepared by the arc discharge method with a Co/Ni (0.6/0.6 atm) catalyst with helium at a total pressure of 500 Torr, with the arc generated by a current of 200A/20V [22], [23]. After synthesis, an indispensable as-grown SWCNT purification (approximately 95%) process was performed. One gram of SWCNTs was refluxed with 3 M HNO3 at 120°C for 32 h, centrifuged at 7,000 rpm, and washed with distilled water in order to purify the SWCNTs. Nitric acid oxidation of the carbon nanotubes exerted a dual role: this treatment was performed in order to decorate the SWCNT surface with -COOH groups and to result in short SWCNTs (length, 50–500 nm). The final solution was dried for 12 h in an oven at 60°C. Finally, 0.75 g of highly pure COOH-SWCNTs was obtained. Subsequently, SWCNTs were fluorinated to a C:F ratio of approximately 2.4∶1 by direct fluorination at 150°C according to a previously reported procedure [24].http://pubs.rsc.org/en/content/articlehtml/2005/cc/b509257d - cit14.

The fluoronanotubes were functionalized with a reaction with the appropriate amine in the presence of a base catalyst. Fluoronanotubes (approximately 15 mg) were sonicated in dimethylformamide (30 mL) for 10 min, resulting in complete dispersion and the formation of a dark solution. A solution of 2-aminoethanethiol hydrochloride (200 mg) in dimethylformamide (20 mL) and 4–5 drops of pyridine (catalyst) was then added. The reaction mixture was stirred (under N_2_) for 3 h at 90°C. The reaction mixture was filtered through a 0.2-µm Cole Parmer Teflon membrane and washed with acetone to ensure complete removal of unreacted 2-aminoethanethiol, reaction by-products, and solvent. The thiol-SWCNTs (SWCNT-SH) were dried overnight in a vacuum at 70°C.

### Retinal Explants and LDH Assays

Whole retinas were carefully dissected and gently placed in culture dishes containing explant medium. This medium was prepared with 1× Basal Medium Eagle, 5% bovine fetal serum, and 1% glutamine. Retinal damage was assessed by measuring LDH that was released in the explant medium, which was expressed as a percentage of total LDH released, as reported previously [25]. LDH is an enzyme that catalyzes the conversion of pyruvate to lactate with concomitant interconversion of nicotinamide adenine dinucleotide hydrogen and nicotinamide adenine dinucleotide. This methodology is usually associated with studies that are conducted on both cardiac and striatal striated muscle. In the nervous tissue, the quantification of LDH that was released into the culture medium was used as an indicator of cell viability [26].

Chick retinal explants (n = 6) were conditioned in the culture medium for 4 h at room temperature. After 1, 2, or 4 h, 400 µL of the culture medium was collected and centrifuged at 100×*g* for 5 min. LDH was measured in aliquots of supernatant with colorimetric quantification with a specific biomedical kit (Labtest Diagnostica SA, Lagoa Santa, MG, Brazil). The results were obtained by measuring light absorbance at 500 nm with a spectrophotometer.

### TUNEL Assays

One-day-lesioned chick eyes were fixed for 30 min in 4% PFA in PBS (0.1 M; pH, 7.3) and cryoprotected in 30% sucrose solution for 24 h at 4°C. After embedding in OCT compound, they were cut transversally (12 µm) on a cryostat, and 1 in 5 sections were sampled from the nasal to the temporal direction, as previously described [27], [28]. For the analysis, we examined 3–5 sections in each animal (n = 3) at the lesioned sites or at the corresponding locations in the control eyes. Labeling was accomplished by incubation in TdT buffer for 10 min at room temperature, followed by incubation with biotinylated dUTP (Roche Molecular Biochemicals, Mannheim, Germany). The reaction was terminated in stop-reaction buffer (10%, 0.1 M ethylenediaminetetraacetic acid, pH 8, in water) and washed in PBS. Counterstaining of the retinas was performed with 4′,6-diamidino-2-phenylindole (DAPI) by incubating the sections at room temperature for 10 min. The sections were mounted with a ProLong Antifade kit (Life Technologies Corporation).

### LCM Cell Asorting and Polymerase Chain Reaction (PCR) Analyses

LCM of stained cryostat sections has been used to separate distinct cell populations to determine specific gene expression patterns in tissues with a complex mixture of cell types [29]. However, RNA degradation is a major drawback in most common fixation protocols recommended for tissue processing prior to LCM [30]. Therefore, specific fixation and RNA isolation protocols have been developed for LCM [17], [31]. We demonstrated the use of this technology with chick retinas, which were dissected (n = 3), fixed in 2% PFA for 15 min, and then incubated with 30% sucrose for 1 h. Cryostat sections (5–10 per retina; 10–12 µm thick) were obtained in the nasal to temporal direction and rapidly stained with cresyl violet.

To examine gene expression in specific cell populations, we performed LCM (LaserScissors 390/20, Cell Robotics International, Inc., Albuquerque, NM, USA) in chick retinal sections. After selection of the region with the laser, different procedures can be employed to extract the tissue from the microscope slide. Herein, to isolate the microdissected cells, the laser was focused on the microscope slide. A single shot at maximum laser power provides sufficient energy to completely detach the isolated fragment, which is collected by electrostatic attraction by a thin plastic coverslip that is placed on top of the specimen without direct contact. With this procedure, fragments of the inner nuclear layer were microdissected from transverse retinal slices (n = 3), and attempts were made to avoid contamination from other layers [29], [32]. RNA was isolated with TRIzol® according to the instructions provided by the manufacturer (Life Technologies Corporation) and the specific changes described in previous studies [17]. Total RNA was treated with 20 units of DNase I (GE Healthcare Biosciences, Piscataway, NJ, USA) to eliminate residual DNA. mRNA was reverse transcribed with the SuperScript II (Life Technologies Corporation) standard protocol. End-point PCR was conducted with primers for chick glyceraldehyde 3-phosphate dehydrogenase (GAPDH; forward: 5′-GGAGCGTGACCCCAGCAACA-3′; reverse: 5′-ACAGCTTCCCAGAGGGGCCA-3′).

### Statistical Analysis

For the LDH experiments employing SWCNTs, we used two-way analysis of variance (ANOVA) with the factors of *time* and *treatment*, which was followed by pair-wise comparisons with Tukey’s HSD test. When we compared LDH release from control vs. lesioned retinas and the results of TUNEL analyses, we used paired *t*-tests.

## Results

### Examining the Injured Retina

**Figure 2:**
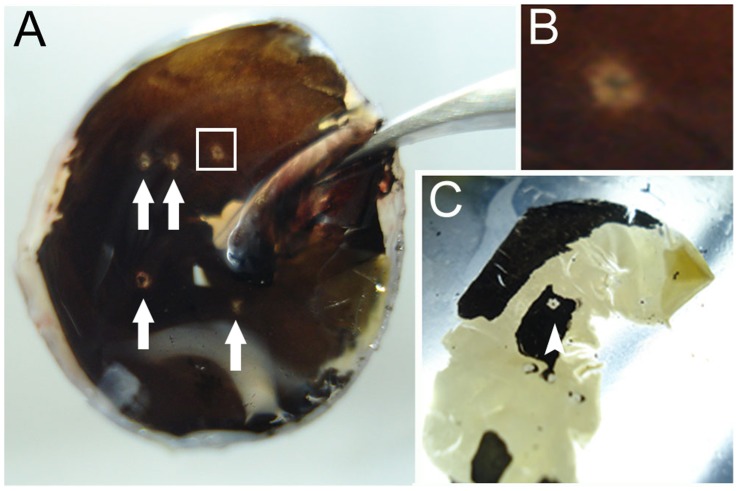
Identification of focal lesions in the chick retina. Mechanical lesions were produced with a thin insulin needle that crossed the cornea, lens, vitreous, retina, and epithelium. (A) After removing the vitreous, it was possible to identify the focal lesions in the middle retina (arrows), close to the *pecten oculi*. (B) High magnification of the selected area in the white square in A is shown. Although there is a semitransparent aspect to the retina, the lesion borders are well defined because the pigmented epithelium was also perforated. (C) Even after the complete removal of the retina from the sclera, it was possible to visualize the lesions containing some surrounding epithelium (arrowhead). Complete removal of the pigmented epithelium is facilitated by incubating the retina in cold phosphate-buffered saline.

Due to its size, eyecups were easily removed from the chick head. When we employed rats, the eyes were cut off from the head with small tweezers. After detaching the vitreous, visualization of the focal lesions in the retina was facilitated with magnifying glasses. As shown in Fig. 2, it was possible to observe the focal lesions right after the dissection procedure. Removal of pigmented epithelium was accelerated by incubating the retina in cold PBS. The lesions were well defined and surrounded by epithelial infiltration. Even after complete dissection of the retina from the eye, focal lesions were observed in the middle of the retina, which contained some epithelium (Fig. 2).

When the retina is processed for quantitative methods, such as real-time PCR or western blotting, the pigmented epithelium should be completely removed.

### Reactive Gliosis within the Focal Lesion

**Figure 3:**
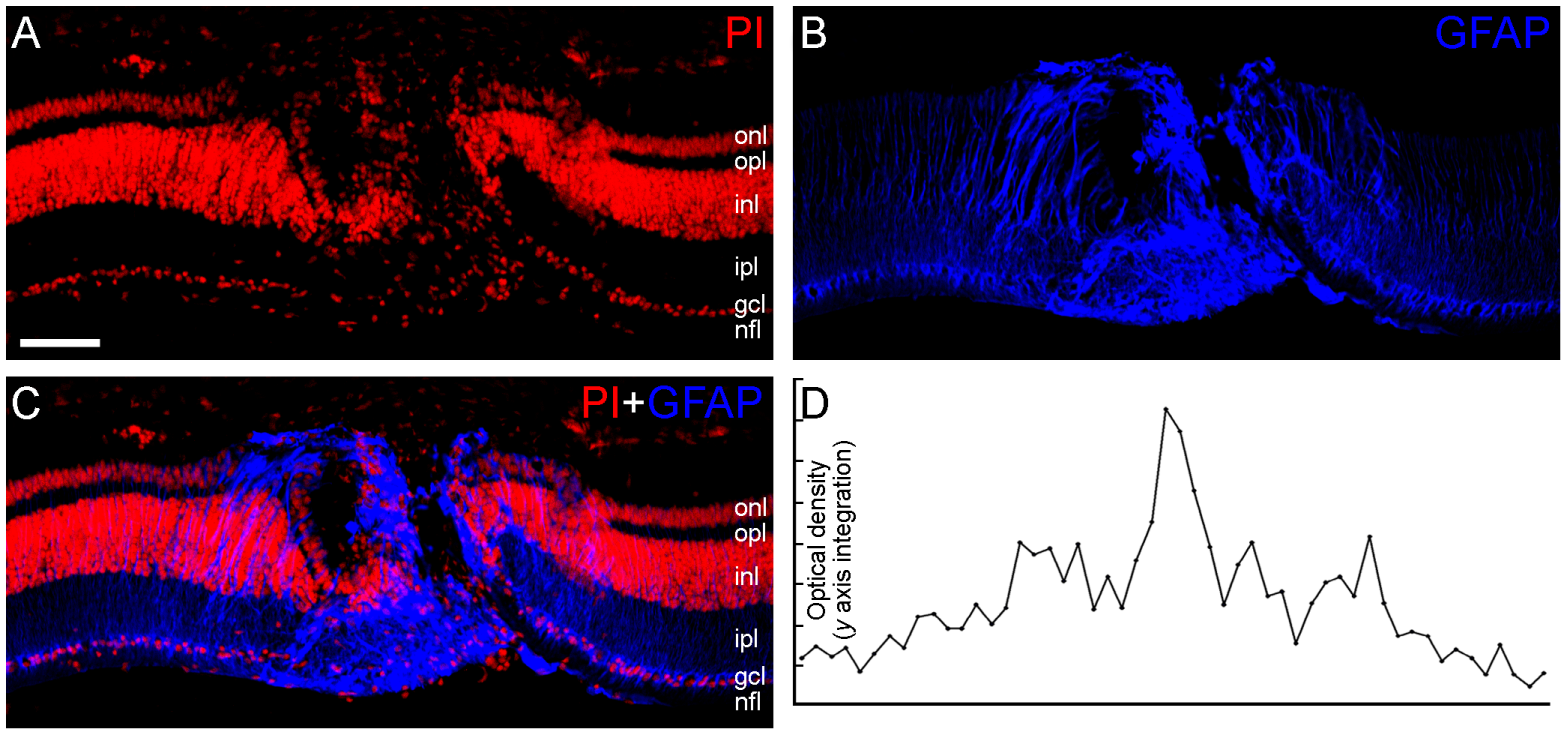
Glial fibrillary acidic protein (GFAP; blue) immunolabeling in vertical sections of 7-day-lesioned chick retinas counterstained with propidium iodide (red). (A–C) Vertical sections of chick retina were counterstained with propidium iodide (red) to facilitate visualization of the nuclear layers. In order to validate the focal lesion model, GFAP (blue) immunolabeling was conducted. We detected typical intense GFAP labeling, representing the reactive gliosis process, which decreased toward the penumbra and adjacent areas (n = 3, 3–5 samples per retina). (D) With the use of a pixel analysis, quantification of the optical density along the focus, penumbra, and adjacent areas was possible. The labels indicate the approximate location of the outer nuclear layer (onl), the outer plexiform layer (opl), the inner nuclear layer (inl), the inner plexiform layer (ipl), the ganglion cell layer (gcl), and the nerve fiber layer (nfl). Scale bar: 60 µm.G.

Differences in immunolabeling can be detected in microscope sections, but variations in the immunolabeling can be misleading. Therefore, the presence of affected and nonaffected regions within the same section is a reliable way to compare specific changes in protein distribution. We performed immunofluorescence assays to evaluate reactive gliosis in the focus of the lesion and the gradient in the surrounding areas (Fig. 3) and we demonstrated that changes in protein distribution that are caused by the neurodegenerative process can be assessed with the proposed model. Therefore, GFAP distribution was analyzed in vertical sections of 7-day-lesioned chick retinas. Chick retinal slices were counterstained with propidium iodide to facilitate the visualization of the nuclear layers [33]. As shown in Fig. 3, this model allows for the spatial definition of the lesion with high reproducibility, permitting visualization of the focus, the penumbra, and the adjacent areas. As expected, within the lesion focus, we observed intense GFAP labeling due to reactive gliosis (n = 3, 3–5 samples per retina). With software that is dedicated to image analysis, it is possible to determine the optical density within the focus, penumbra, and adjacent areas (Fig. 3).

### LDH Release is a Reliable Probe of Retinal Cell Viability

**Figure 4:**
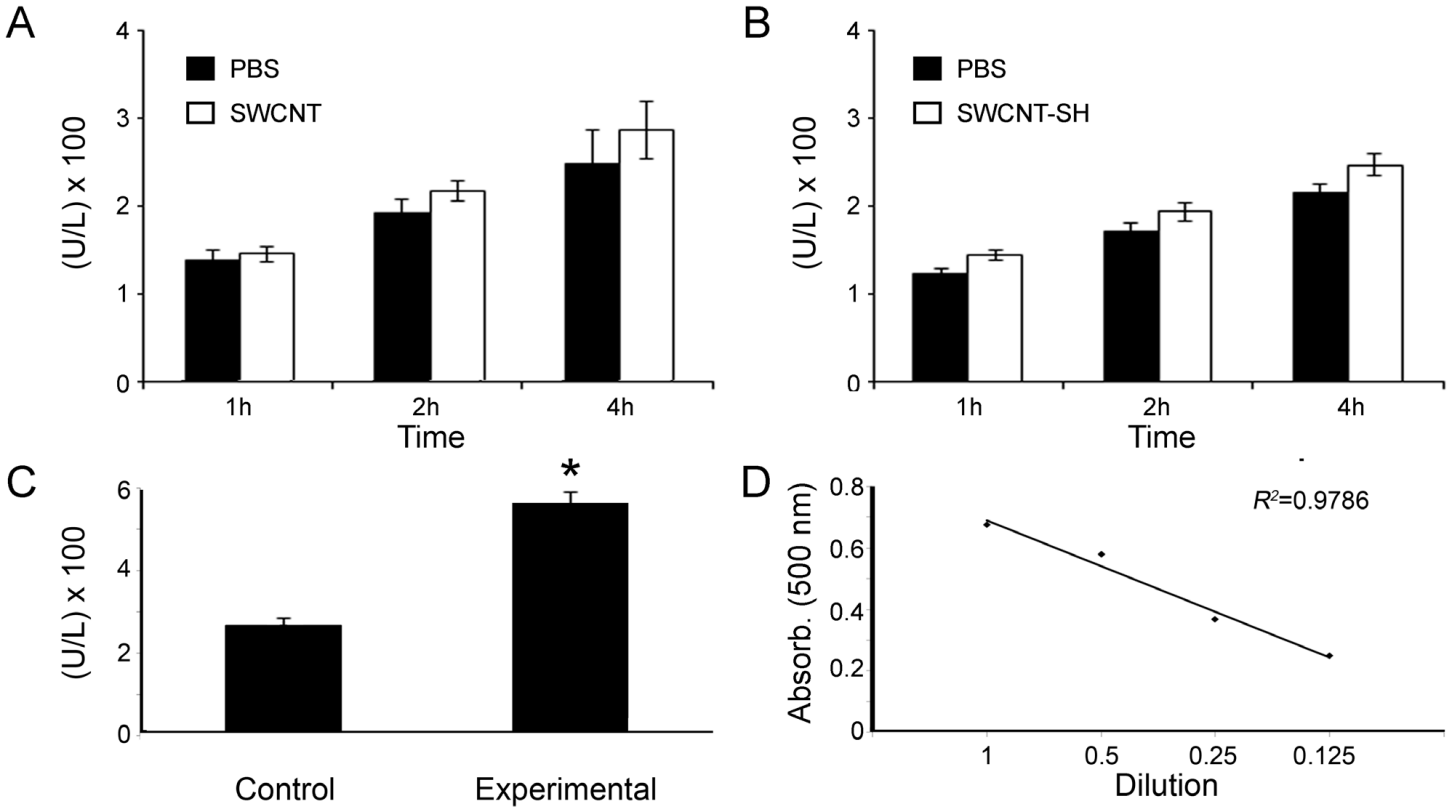
Quantification of cell viability with a lactate dehydrogenase (LDH) assay in rat retinal explants. We determined the effects of two distinct carbon nanotube compounds on retinal explants, (A) single-wall carbon nanotubes (SWCNT) and (B) thiol-single-wall carbon nanotubes (SWCNT-SH). LDH accumulated in the explant medium over time. Thus, we observed crescent values of absorbance (500 nm) after 1, 2, and 4 h. We did not detect significant differences in LDH concentrations that were released by undamaged retinas incubated with the two distinct nanotube compounds (10 µg/mL). (C) As a positive control, we compared the concentrations of LDH that was released from 1-day-lesioned *vs.* nonlesioned retinal explants. As expected, lesioned retinas released more LDH (n = 6, *P<0.01). (D) Explant medium was diluted in different concentrations and tested in an LDH assay. A linear regression analysis of the plots generated from medium serial dilutions ranging from 100% to 25% revealed high correlations between the concentrations and the readouts (R^2^ = 0.9786), indicating detection linearity.

As an example of LDH assay employment in neurodegeneration studies, we tested the effects of carbon nanotubes on concentration of LDH released by undamaged rat retinas. LDH is released in explant medium over time, allowing for the measurement of its concentrations at different time points. As expected, we observed increasing absorbance values after 1, 2, and 4 h (Fig. 4). However, we did not detect significant differences in concentrations of LDH released from explants incubated with two distinct nanotube compounds (10 µg/mL). As a positive control in the experiment, we compared concentrations of LDH released from 1-day-lesioned *vs.* nonlesioned rat retinal explants. Lesioned retinas released more LDH after 4 h in culture (n = 6, *P*<0.01). To test detection linearity, 4-h explant medium was diluted to different concentrations and subjected to an LDH assay. Linear regression analyses of plots generated from medium serial dilutions ranging from 100% to 25% revealed a high correlation (R^2^ = 0.9786), indicating detection linearity (Fig. 4). Taken together, these results indicated that LDH is a reliable probe for retinal cell viability *in vitro*.

### LCM Cell Sorting in the Retina Followed by PCR Analysis

**Figure 5:**
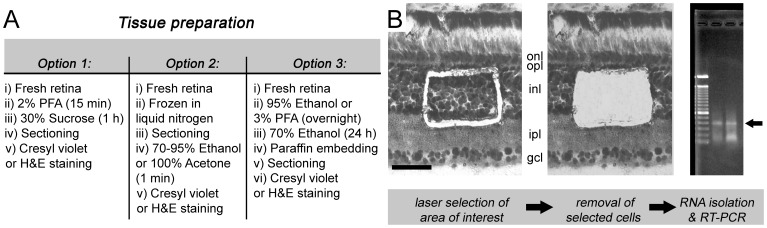
Laser capture microdissection (LCM) followed by reverse transcription-polymerase chain reaction (RT-PCR) allows for quantitative gene expression analyses of specific cell populations. (A) Different protocols were described to prepare samples for LCM cell sorting, including (i) fixation procedures for sectioning with a cryostat, (ii) sectioning that is followed by fixation *in specimen*, and (iii) preparation for paraffin embedding. Microdissection of the inner nuclear layer in 10–12 µM chick retina cryostat slices exemplifies the use of this technique. (B) The retina was rapidly counterstained with cresyl violet to permit the identification and isolation of a specific retinal layer. In order to isolate the microdissected cells, the laser was focused on the microscope slide. A single shot at maximum laser power provides sufficient energy to completely detach the isolated fragment, which is collected by electrostatic attraction to a thin plastic coverslip that is placed on top of the specimen. RNA isolation was performed with protocols that were modified from conventional commercial kits. After 35 cycles of conventional PCR, we detected specific bands for chick glyceraldehyde 3-phosphate dehydrogenase (GAPDH; arrow). In addition to fresh tissue, this technique can be performed in retinal explants. Scale bar: 60 µm. Partial reproduction from our previous work [44].

We prepared samples for LCM to select fragments from a specific retinal layer. Microdissected cells from the inner nuclear layer of the chick retina were isolated and carefully harvested to perform RNA isolation, cDNA synthesis, and PCR analysis. We were able to detect GAPDH bands at the expected molecular weight, confirming the mRNA integrity (Fig. 5). The importance of this result is two-fold. First, these results showed that the retrieval of mRNA could be achieved in the fixed retina, as described in previous studies. Moreover, these results disclosed that it is possible to assess changes in gene expression during neurodegeneration in specific neuronal cell types.

### Evaluation of Apoptotic Dynamics

**Figure 6:**
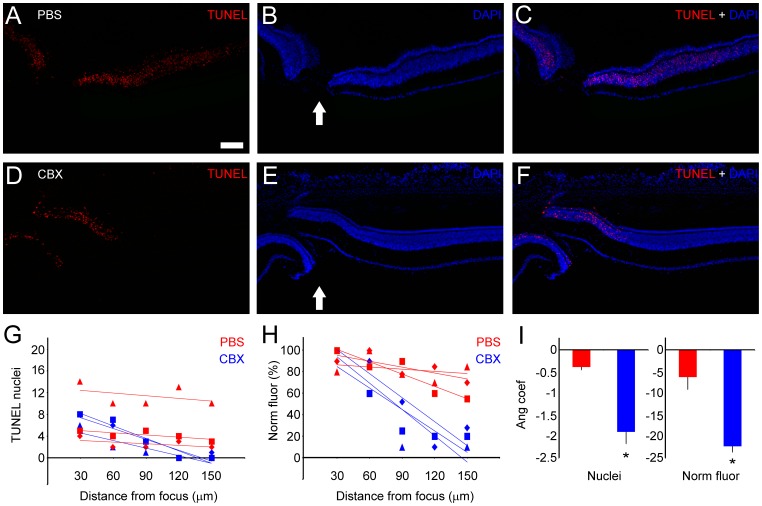
Modeling apoptotic spread in degenerating tissue. Explants of 1-day-lesioned chick retinas were maintained for 4 h in culture media with (A-C) phosphate-buffered saline (PBS) or (D-F) 100 µM of carbenoxolone (CBX). Afterwards, transverse sections were submitted to terminal deoxynucleotidyl transferase (TdT)-mediated 2-deoxy-uridine-5-triphosphate (dUTP) nick-end labeling (TUNEL) in order to characterize the apoptotic spatial pattern. In each image, it was possible to localize the focus of the lesion (arrows). (G) In order to determine whether gap junction blockers caused changes in the distribution of apoptotic cells, we counted the number of TUNEL-positive nuclei that were located as far as 150 µm away from the focus of the lesion. The values were plotted according to the distance from the focus, and they were subjected to linear regression with the least squares approach, generating mathematical parameters, such as *R^2^* and *R*, and the first-order equation (*y = ax+b*). (H) The same procedure was undertaken with values from the pixel analysis. An *x–y* axis bitmap analysis was used to view the pixel values in numeric format, in which the values corresponded to the brightness of the pixels. Considering the distribution of TUNEL-positive nuclei, the means of the angular coefficient from the first-order equation (*a*) were calculated for each experimental condition (n = 3). (I) Compared with the control (−0.40±0.06), the absolute value of *a* was higher with CBX treatment (−1.83±0.35, P<0.05). With regard to the values from the bitmap pixel analysis, we detected significant changes in the *a* angular coefficient when comparing PBS (−6.33±2.77) vs. CBX (−22.3±1.3, P<0.05) treatments. *P<0.05 in a paired *t*-test. Scale bar: 60 µm.

As we have shown, an LDH assay may provide a quantitative measure of cell viability, which reflects the integrity of neuronal membranes. However, the apoptotic process is classically related to fragmentation of the cell nucleus, and detection of this particular condition is frequently performed with a TUNEL assay [27]. Although TUNEL has been shown to be a reliable method for the detection of apoptotic cells, analyses of TUNEL labeling do not have suggested standards. Thus, fine and precise evaluations of prospective neuroprotective effects could be quite difficult. To overcome this hindrance, we developed a simple and accurate method to model apoptosis spread (Fig. 6).

Herein, as an example, we evaluated the effects of the gap junction blocker carbenoxolone (CBX) on the distribution of apoptotic cells in chick retinal explants. One-day-lesioned retinas were maintained in culture for 4 h at room temperature. By taking the lesion focus as a reference, the distribution of apoptotic nuclei was typically narrower in retinas that were treated with CBX (100 µM), although some variations in spatial distribution were observed. To test the consistency of this result, we employed two different quantification methods. First, we plotted the number of apoptotic nuclei that were counted at different distances from the lesion focus. Next, data plots were submitted to a linear regression with the least squares approach, which generated parameters, such as *R^2^* and *R*, and a first-order equation (y = ax+b) for each experimental condition. As a second approach, the values from bitmap analyses were submitted to the same procedure. The bitmap analysis was used to view the pixel values of active windows in numeric format, in which the values corresponded to the brightness of the pixels. The mean of the angular coefficient from the first-order equation (*a*) was calculated for each experimental condition (n = 3) as a parameter of apoptotic spread (Fig. 6).

For the number of apoptotic cells, the absolute value of *a* was higher with CBX treatment (−1.83±0.35, P<0.05) compared with PBS treatment (−0.40±0.06). With similar procedures, we were able to detect significant changes in the *a* values that were obtained from the bitmap pixel analysis comparing PBS (−6.33±2.77) vs. CBX (−22.3±1.3, P<0.05) treatments (Fig. 5). Taken together, these results revealed that apoptotic spread could be compared and quantified with a combination of *in vivo* lesions, *in vitro* treatments, and mathematical analyses. The effects of CBX on apoptotic spread and the presence of gap junction proteins in dying neurons have been previously reported by our group [28].

## Discussion

Immunohistochemistry has been a valuable tool in both the diagnosis and research of infectious and degenerative diseases [34], [35]. Differences in immunolabeling can be detected in microscope sections, but these variations can be misleading. In this regard, the presence of experimental and control regions within the same section is a reliable way to compare specific changes in protein distribution in a degenerative tissue. As we demonstrated, the proposed model of retinal degeneration allowed for the visualization of the focus, the penumbra, and the adjacent areas within the same image. As an example, we were able to detect undisputed changes in GFAP labeling, which characterize the process of reactive gliosis. Indeed, gliosis is a hallmark of neurodegenerative conditions in several regions, including the retina [36]. GFAP immunolabeling spanned all the retinal layers within the lesion focus and decreased toward the penumbra areas. In adjacent areas, GFAP labeling was similar to that seen in nonlesioned retinas. Müller glial cells undergoing reactive gliosis are believed to alter the local environment of neurons and provide additional structural integrity to the retina at the site of injury by inducing changes in intermediate filament constituents [37].

In addition to gliosis, another common feature in the neurodegenerative process is that secondary cell death is frequently more detrimental than the loss caused by the first injury. Pharmacological agents have been tested in an attempt to decrease the apoptotic program and/or spread, despite the fact that the balance of the benefits and detriments of these strategies remains under debate [38], [39]. In this regard, *in vitro* approaches are quite useful for testing the efficacy of specific molecules. Indeed, neuronal, glial cell, and mixed cultures offer the convenience of *in vitro* models, but the disruption of the original organization and the lack of the extracellular matrix prevent a more realistic and pathophysiological interpretation of the data. With the use of retinal explants, cells retain many histological and biochemical features and can be maintained for several days or even weeks [1], [40]. It has been reported that long-lasting neuronal cell cultures remain viable for months, thus adding a few easy procedures to traditional protocols [41]. However, although the time in culture cannot be compared, explants have an important advantage: they are relatively easy to establish because the tissue is directly transferred to a culture plate containing explant medium following the dissection. However, shorter periods in culture have additional advantages. An optic nerve axotomy does not severely affect ganglion cell survival in the time scale that we evaluated in culture [42]. Similar effects were observed in other regions of the nervous system [43], indicating that neurons remained viable for shorter periods after axotomy.

As we demonstrated, an LDH assay can be performed with the explant medium after different time points in culture. The measures of the LDH amounts allow for an evaluation of cell membrane stability, which in turn reflects cell viability. As expected, we observed that LDH release was higher in 1-day-lesioned retinas compared with that in the respective controls. In fact, previous retinal studies have reported that secondary cell loss occurs predominantly by apoptosis and mostly within 24 and 36 h [13]. LDH assays are highly compatible with other contemporary approaches that are based on RNAi, antagomirs, aptamers, and overexpression systems and aim to examine neuroprotection. Furthermore, retinal explants could be useful for testing newly developed techniques of drug delivery, such as those that are combined with peptide or carbon nanotubes [9], [10].

After the role of a particular molecule in cell death/survivor is determined, *in vivo* testing can be performed with intraocular injections. In this case, presumably neuroprotective effects can be verified after acquiring data provided by *in vitro* observations. Again, this has several advantages, such as easy access to the retina, a well-known morphology, and the fact that both experimental and control conditions can be tested in the same animal.

In summary, the combination of focal lesions in the retina and an *in vitro* approach permits the use of several distinct and contemporary techniques, which can be quite useful in studies examining neuroprotection.
