# Correction: A New and Reliable Guide for Studies of Neuronal Loss Based on Focal Lesions and Combinations of *In Vivo* and *In Vitro* Approaches

**DOI:** 10.1371/annotation/4744b3c3-13a8-452d-9c59-5ca80c9614fe

**Published:** 2013-06-21

**Authors:** Vera Paschon, Guilherme Shigueto Vilar Higa, Lais Takata Walter, Érica de Sousa, Fausto Colla Cortesão Zuzarte, Vivian Roca Schwendler Weber, Rodrigo Ribeiro Resende, Alexandre Hiroaki Kihara

The name of the fourth author was spelled incorrectly. The correct name is: Erica Sousa. The correct citation is: Paschon V, Higa GSV, Walter LT, Sousa E, Zuzarte FCC, et al. (2013) A New and Reliable Guide for Studies of Neuronal Loss Based on Focal Lesions and Combinations of In Vivo and In Vitro Approaches. PLoS ONE 8(4): e60486. doi:10.1371/journal.pone.0060486. The correct abbreviation in Contributions is: ES.

